# 28.1 VARIETIES OF SELF DISORDER: A BIO-PHENO-SOCIAL MODEL OF SCHIZOPHRENIA

**DOI:** 10.1093/schbul/sby014.116

**Published:** 2018-04-01

**Authors:** Barnaby Nelson, Louis Sass

**Affiliations:** 1 Orygen Youth Health Research Centre; 2 Rutgers University

## Abstract

**Background:**

The self-disorder model offers a unifying way of conceptualizing schizophrenia’s highly diverse symptoms (positive, negative, disorganized), of capturing their distinctive bizarreness, and of conceiving their longitudinal development. These symptoms are viewed as differing manifestations of an underlying disorder of ‘core-self’: hyperreflexivity/diminished-self presence with accompanying disturbances of “grip” or “hold” on reality.

**Methods:**

We have recently revised and tested this phenomenological model, in particular distinguishing primary versus-secondary factors, in offering a bio-pheno-social model of schizophrenia spectrum disorders.

**Results:**

The revised model is consistent with recent empirical findings and offers several advantages:

1) It helps account for the temporal variations of the symptoms or syndrome, including longitudinal progression, but also the shorter-term, situationally-reactive, sometimes defensive, and possibly quasi-agentive variability of symptom-expression that can occur in schizophrenia (consistent with understanding some aspects of self-disturbance as dynamic and mutable, involving shifting attitudes or experiential orientations).

2) It accommodates the overlapping of some key schizophrenic symptoms with certain non-schizophrenia spectrum conditions involving dissociation (depersonalization and derealization), including Depersonalization Disorder and Panic Disorder, thereby acknowledging both shared and distinguishing symptoms.

3) It integrates recent neurocognitive, neurobiological, and psychosocial (e.g., influence of trauma and culture) findings into a coherent but multi-factorial neuropsychological account.

**Discussion:**

An adequate model of schizophrenia will postulate shared disturbances of core-self experiences that nevertheless can follow several distinct pathways and occur in various forms. Such a model is preferable to uni-dimensional alternatives—whether of schizophrenia or core self disturbance—given its ability to account for distinctive yet varying experiential and neurocognitive abnormalities found in research on schizophrenia, and to integrate these with recent psychosocial as well as neurobiological findings.

